# Law’s Image of the Human

**DOI:** 10.1093/ojls/gqaa026

**Published:** 2020-10-17

**Authors:** Gustav Radbruch

**Keywords:** jurisprudence, legal history, legal philosophy, natural law, legal reasoning

## Abstract

This is a translation of ‘Der Mensch im Recht’, Gustav Radbruch's inaugural lecture at the University of Heidelberg in November 1926, translated with an Introduction by Valentin Jeutner. Radbruch addresses the way in which law's image of the human informs the operation and content of law.

## Translator’s Introduction (Valentin Jeutner)

At around 12 o’clock on Saturday, 13 November 1926, Gustav Radbruch walked down a narrow aisle between rows of chairs towards the cathedra of Heidelberg University’s Great Hall. Left and right, he passed university officials, professorial colleagues, students and members of the public. Some of them might have seen the headlines in Heidelberg’s newspapers that morning. The *Heidelberger Tageblatt* reported that Germany’s minority government of Chancellor Wilhelm Marx (Catholic Centre Party) had been saved, for now, by the Social Democrats.^1^ The *Heidelberger Neueste Nachrichten* proclaimed that French–German relations were steadily improving.^2^ A national daily, the *Deutsche Allgemeine Zeitung*, noted that one of the last ‘Black Reichswehr’ trials concluded with acquittals and mild sentences for members of the paramilitary group which had attempted to overthrow the democratic German government in 1923.^3^ These headlines capture quite accurately the prevailing spirit within the Weimar Republic, which continued to struggle in the aftermath of the perceived humiliation at Versailles and was attempting to re-establish itself as a respectable member of the community of states. For a while, Radbruch himself had been at the front line of this struggle, serving as a member of the constitutional convention at Weimar (1919–1920), as a member of the Reichstag (1920–1924) and twice as a social-democratic Minister of Justice (1921–22, 1923). However, having discovered that he was more of a ‘contemplative rather than an active type’,^4^ Radbruch consciously decided to return to academia full-time: first to Kiel and, eventually, in 1926, to Heidelberg.

As Radbruch entered the Great Hall to deliver his inaugural lecture as Heidelberg University’s newly appointed Professor of Criminal Law, his surroundings would have been quite familiar to him. Prior to the First World War and his political excursions, he had resided in Heidelberg from 1904 to 1914, completing his most important work—*Outlines of Legal Philosophy*^5^—during that time. In the common law world, Radbruch’s *magnum opus* is overshadowed by the fame of his 1946 article entitled ‘Statutory Lawlessness and Supra-Statutory Law’.^6^ The article considered the relationship between law and morality, and subsequently formed part of a controversy which is now known as the Hart–Fuller debate.^7^ A key aspect of that debate concerned the way in which human legal subjects relate to law.^8^

In his lecture, Gustav Radbruch decided to focus on the opposite of that relation—namely, on the question of how law relates to its human subjects. As Radbruch explains in the lecture’s first sentences, his focus is not on how individuals relate to law, or on the actual or ideal characteristics of human individuals. Instead, Radbruch wants to investigate law’s legal image of those human individuals to whom law is addressed.

Radbruch maintains that this legal image of the individual keeps changing. He illustrates his argument by considering law’s image of the individual from medieval German law up to the legal system of Radbruch’s time. One contemporaneous commentator critically observed that Radbruch’s overview of how law’s image of the human changed over time remains broad and is insufficiently detailed.^9^ This observation is correct to the extent that Radbruch’s lecture will disappoint anyone who is looking for a systematic exposition of the way in which law related to the individual throughout the last 500 years across all of law’s sub-disciplines. However, Radbruch’s general approach to jurisprudence is hardly ever informed by a desire to offer a comprehensive doctrinal treatment of specific questions.^10^ Rather, his approach is characterised by identifying and engaging fundamental questions of law in a manner that allows for a critical reflection upon the reasons that inform a given (legal) state of affairs. In this regard, his inaugural lecture is no exception.^11^ Of the many observations he makes in his lecture, three of particularly enduring importance shall be highlighted and contextualised briefly here.

The first point relates to Radbruch’s claim that it is law’s image of the individual that shapes the character of a legal epoch. Radbruch claims that ‘Nothing defines … the character of a legal era more clearly than the conception of the individual upon which it relies’. In other words, law’s image of the individual reflects law’s character. In this way, Radbruch explicitly extends Georg Jellinek’s claim that any theory of the state departs from a particular understanding of Adam, humanity’s supposed progenitor, to the realm of law.^12^ At the same time, he foreshadows more recent variants of that argument advanced, for example, by Alain Supiot.^13^ Radbruch’s connection of a legal era’s character with an era’s image of the individual is significant since it renders concrete the otherwise abstract image of the character of a legal era and since it offers an easily accessible point of departure for a systematic analysis of a given legal order. With respect to the first aspect, Radbruch’s argument renders the image of the character of a legal era concrete by shifting the observer’s focus away from general questions about the state of law to law’s subject. This shift means that it is not law that appears as history’s self-serving agent, requiring individuals as human subjects for the purpose of implementing law’s commands or exercising law’s rights. Instead, law emerges as no more than a manifestation of the acts and intentions of human actors.^14^ As a result, it becomes more difficult to treat the abstract image of law as an independent entity. Any legal analysis must be related to its human subjects. With respect to the second aspect, this concretisation of a legal era’s character provides a straightforward method to reconstruct the character of a particular legal system by assessing upon which image of an individual subject a legal era relies. Instead of studying a projection of countless pixels on a cinema screen, Radbruch argues one could learn just as much, if not more, about the projection by focusing instead on the projector (the characteristics of the person which condition the shape of the legal order). It should be noted that Radbruch does not claim that one can reconstruct the character of a legal order from an ‘empirically concrete person’; instead, he speaks throughout of law’s (legal) image of the person to whom it relates. This becomes particularly apparent when considering the second point below.

Having outlined this first general observation, Radbruch proceeds to explain just how law’s image of an individual could be found. He argues that the best method is not to solely study how legal provisions define legal concepts like ‘individual’, ‘person’ and ‘human’, but rather to consider ‘the subjective rights and the legal duties a particular legal order has fashioned’. Radbruch’s underlying assumption is that a legal order creates rights where human impulses are aligned with the legal order’s intentions, and that it creates obligations where the legal order anticipates human impulses that would frustrate the legal order’s intentions. In this way, the characteristics of law’s individual subject can be inferred from the study of the subjective rights and obligations imposed by law and vice versa. Again, it should be remembered that Radbruch considers the individual from a legal point of view, and that this proposed method identifies merely the contours of the legal mould—the imprint that the image of the individual leaves upon the legal order. While Radbruch’s lecture does not aim for an evaluation of the discrepancies between the actual human and the legal human,^15^ his proposed method of identifying law’s image of the individual in the more abstract legal sense still allows for a critical engagement with the assumptions that inform law’s image of the individual. This is so because the consideration of the legal mould of the characteristics of an individual also makes immediately apparent which characteristics, and which types of humans, do not fit into that mould. For example, it becomes possible to determine if a legal system is tailored to the characteristics and needs of male members of a property-owning class or if it also recognises characteristics of other members of society. The lively debates in the common law world concerning the more or less discriminatory characteristics of the so-called reasonable man illustrate the efficacy of analysing law from the point of view of an imagined, individual legal subject very well.^16^ Apart from enabling a critical evaluation of the extent to which law’s image of an individual is constructed in inclusive/exclusive terms, analysing law via the image of the individual also has some fundamental benefits, which will be explored in the next section.

The lecture’s third observation of enduring importance relates to Radbruch’s determination to maintain a close connection between law and the human. It appears to be the case that Radbruch’s concern in this regard is informed by at least two convictions: one more instrumental and the other more idealistic in nature. Radbruch’s instrumental concern is that a legal order will break down if the discrepancy between that legal order’s image of the individual human and the characteristics of the average ‘empirically concrete human being’ grows too large. Accordingly, Radbruch argues that the medieval German Reich ‘collapsed owing to its reliance on a conception of the individual that had become more and more inaccurate’. Considering, re-evaluating and, if necessary, adjusting law’s image of the individual thus ensures that law does not lose touch with the social reality to which it aims to relate.

However, Radbruch did not merely think that it is helpful to ensure that law’s image of the individual remains somewhat linked to the actual individual in order to maintain order. He also thought that it is normatively desirable that law is understood and studied with reference to the individuals whose lives it orders. In the lecture itself, Radbruch merely appears to describe the emergence of a new image of the human which is no longer a self-centred individualist but a *Kollektivmensch* who is an integral member of society.^17^ But as his subsequent writings^18^ and the observations of the majority of contemporary commentators demonstrate,^19^ Radbruch fundamentally believed that this alleged development was a positive one that served the interests of humanity. For some, Radbruch’s lecture even marked the beginning of Radbruch’s turn towards a new legal philosophy committed to social welfare,^20^ and dedicated to the ‘humanum’.^21^ This turn eventually culminated in the new edition of his *Rechtsphilosophie*, published in 1932, where Radbruch explicitly related law’s individual subject to the principles of justice, purposiveness and legal certainty.^22^ The way in which Radbruch places the individual in the centre of attention clearly differs from that of Hart, for example, whose theory of law is content-neutral in the sense that it can be applied with ease to a society of ‘deplorably sheep-like’ individuals who ‘might end in the slaughter-house’.^23^

Radbruch concluded his inaugural lecture with an expression of gratitude for being able to complete his days at Heidelberg. However, Radbruch’s position soon changed dramatically. The Weimar Republic, whose fragility the Heidelberg newspapers had captured so accurately on 13 November 1926, collapsed and gave way to the reign of the National Socialists. Shortly after Adolf Hitler came to power, Radbruch was one of the first professors to lose their chair at Heidelberg University in 1933. It might be tempting to conclude that the 12 years of totalitarian Nazi rule that followed illustrate the dangers of thinking of the individual as a *Kollektivmensch* whose advent Radbruch had so fondly described in 1926. However, while the national socialist, de-individualised notion of a *Kollektivmensch* explicitly prioritised collective interests at the expense of individual interests, Radbruch’s concept of the *Kollektivmensch* imagined individuals as members of a community explicitly in order to bring about a more humane legal order that would ultimately benefit individuals. Thus Radbruch writes that approaching legal subjects as *Kollektivmenschen* should bring about the emergence of a social law ‘which is tailored not to de-individualised persons deprived of their unique characteristics’, but instead to the ‘concrete human being in its social context’.^24^ In Radbruch’s case, the ‘collective’ is invoked to enhance and complicate the image of the individual person. In the national socialist context, the ‘collective’ is invoked to displace the interests and the individuality of human beings.

After the end of the Second World War in 1945, Radbruch was rehabilitated, and he continued teaching at Heidelberg until 1948, the year he turned 70. It is noteworthy, but maybe not surprising, that many publications on the occasion of Radbruch’s retirement underline the crucial importance of the term *Mensch* for Radbruch.^25^ Thus, the student representative speaking at Radbruch’s farewell ceremony on 13 July 1948 expressed the students’ gratitude for Radbruch having taught them the law ‘not as a dead machinery of co-dependent norms but … as a cultural force in the midst of which stands steadfast the human’.^26^ Radbruch died shortly after, aged 71, on 23 November 1949. In an obituary, published in December 1949, *The Times* listed his inaugural lecture on ‘Law’s Image of the Human’ as one of the works that ‘secure for Radbruch an honourable place in German legal science’.^27^

## *Law’s Image of the Human* ^28^

When I speak about the individual—the individual human being—in the law, my subject is not how law judges the individual, or how law affects the individual, or how law is supposed to affect the individual. Rather, my subject is how law imagines the individual human being whom it aims to affect, the kind of individual for whom law is made. My topic is not the real individual but law’s notion of the individual to whom its orders are addressed.

This image has varied in different legal eras. One might even say: in the history of law, it is the change of the image of the individual that is ‘epoch-making’. Nothing defines the character of a legal era more clearly than the conception of the individual upon which it relies.

A legal order cannot be tailored to the actual, real human being who walks the surface of the earth, to their peculiarities and moods, to their dottiness, to the entire herbarium of strange plants that we call mankind. From the empirically concrete individual human being, the road does not lead to a legal order but to the negation of a legal order. If one, like Max Stirner, begins with the unique ‘individual’,^29^ one can logically, like him, only end with anarchism. A general legal principle must, rather, be geared towards a general type of individual human being—and, for different legal eras, many different human characteristics appear to be typical or essential, or appear to be decisive targets in framing legal norms.

A legal order’s conception of the individual becomes clearly apparent when one considers the subjective rights and the legal duties a particular legal order has fashioned. One may assume that a legal order is just as interested in the exercise of the rights it confers as in compliance with the duties it imposes. Jhering showed convincingly that a legal order must collapse not only when its duties are not complied with, but also when its rights are no longer exercised.^30^ Thus, a legal order’s intention to guide behaviour is expressed both by the rights it confers and by the duties it imposes. Now, when will a legal order express its intention in the form of a right, and when in the form of a duty? The legal order will confer rights where the legal order can assume that individual impulses are aligned with the intentions of the legal order, so that individual impulses facilitate the implementation of the legal order’s wishes. The legal order will impose duties where the legal order believes that individual impulses run contrary to the legal order’s intentions, so that these impulses must be supplemented with reasons to act in accordance with the legal order’s intentions. Accordingly, the rights and duties that a legal order creates clearly reveal which individual impulses the legal order assumes to exist and to be effective.

I can speak of Medieval German law and the image of the individual upon which it is founded only as a layman. To me, this legal era is characterised by the frequency of duty-infused, duty-based rights, of rights that are granted in the expectation that they will be duly exercised. If such rights are to function effectively, they must presuppose the existence of an individual tied to society by customs, religion and duty. Indeed, the economic and governmental order of the Middle Ages was geared towards such a conception of the individual. The regulation of the guilds entailed granting monopolies in the belief that the honour of belonging to the guild was a sufficient incentive to produce high-quality work—and this belief proved amply justified over the centuries. The feudal system meant that rights were to the largest extent granted under the nearly uncontrollable and unenforceable premise that they would be exercised in the spirit of fealty. This premise eventually failed: feudal lords became rulers; the Reich collapsed. Ultimately, the Reich collapsed owing to its reliance on a conception of the individual that had become more and more inaccurate.

The Renaissance, the Reformation and the Reception^31^ had unchained the individual from the community. This individual, unleashed from the community, no longer driven by duty but by self-interest, is law’s point of departure during this time period. This new type of individual in the law is crafted in the image of the merchant who calculates and strives entirely for profit (‘business is business’). The needs of the merchant were one of the crucial causes of the Reception and thus of law’s transition to a new conception of the individual. Since then, one can say without exaggeration that the law conceives of anyone as a merchant: even the worker is thought of as ‘selling labour’.

The legal era that conceives of the individual as personified self-interest breaks down into two time periods: Police State^32^ and Enlightenment. The Police State is still suspicious of the immature mind of the legal actor [*Rechtsgenosse*]. The Police State is prepared and concerned to protect the legal actor against its own errors, to make people happy against their will. The Police State is (in the words of a 1766 *Hofkammerordnung* from Baden) the natural guardian of its subjects, whom the Police State is also prepared to instruct against their will on how they should run their own households. Even where well-understood self-interest already points in the same direction, the Police State frequently creates not only rights, but also duties. That which is not prohibited is prescribed—nothing is merely permitted. The legal order conceives of its addressee as an individual who is sufficiently self-interested to be exclusively driven by interests but insufficiently mature actually to recognise what those interests are.

It was only in the period of the Enlightenment and natural law that the legal order adjusted itself to the type of individual that Roman law presumed to exist: an individual who is not only highly self-centred, but self-centred in a notably prudent manner, an individual who follows well-understood self-interests, is free from all sociological ties and is subject to legal ties only by having consented to be bound by them, in accordance with well-understood self-interests. This image of the individual encapsulates both a permanent methodological insight and a transient, historically conditioned understanding. As to the former, any lawmaker needs to design laws in a manner that takes account of the fact that individuals may be so self-interested as to follow their own interests ruthlessly, except where law prevents them from doing so, and so clever as immediately to spot any loophole in the regulatory framework. The legislator’s laws must even (to echo Kant)^33^ fit a nation of devils, assuming they are intelligent. In the same spirit, Machiavelli observes that ‘those who lay the foundations of a State and furnish it with laws must … assume that all men are bad’.^34^ Accordingly, an old saying provides that ‘bad manners make good law’. Thus, every law must be tailored to, and must be tested by reference to, the fictional construction of a highly self-centred and very clever individual. For this legal era, however, this type of individual human being was more than a fictional construction—it was an empirical average type. Just like classical economics, the contemporary school of natural law also believed that the majority of individuals resembled the image of the *homo oeconomicus*. This light and lively period had a hard time believing that most human beings were not self-centred, understanding and active, but, rather, good natured, ignorant and lazy.

It was only this naive belief in the true existence of its conception of the individual that enabled this era to adjust the entire legal order to the new type of human being, with remarkable consistency. The remains of the medieval-patriarchal legal order disappear: all of the rights that were granted in the illusory expectation of dutiful exercise are now carefully dissolved into special rights and special duties. Similarly, the duties imposed by the Police State in furtherance of the individual, though unperceived, interests of a norm-addressee are removed. Where self-interest already points in the same direction, no duties are imposed. Rather, rights are granted: *beneficia non obtruduntur*; those who have no wish already have what they desire; the individual’s will is his heavenly kingdom. In this regard, the law presupposes both cleverness and activity that recognises interests and the means to realise them. This includes an awareness of the applicable legal means: *ignorantia juris nocet, jus vigilantibus scriptum—*law shows no consideration towards the unwary. All ties, except those imposed by the law itself—all social and economic ties that could impede the pursuit of the individual’s well-understood interests—are ignored. Legal possibility is equated with factual possibility. The formal, legal freedom of contract is, for example, considered to be an actual freedom of contract. All individuals—thought to be equally self-centred, prudent, active and free—are thus thought to be equal. Contracting parties resemble each other, as if they were mirror images. In legal affairs, the same individual confronts its ghostly doppelgängers, thousands of times and in manifold roles.

Until recently, this image of the individual dominated legal thought more than we knew and more than we might have wanted to believe. Originating from private law, this image soon impressed itself upon civil proceedings: the principle of party autonomy means that the proceedings are conducted as if two chess players—two cunning opponents driven by well-understood interests, not in need of judicial assistance—face off against one another. Under Feuerbach, criminal law is dominated by the same image of the individual: Feuerbach’s theory of psychological coercion presupposes individuals who self-centredly and prudently, undistracted by impulses or burdened by conscience, balance the costs and benefits of their intended crime, and then act in accordance with their well-understood self-interest.^35^ In accordance with social contract theory, public law, too, is considered to be founded upon the well-understood particular interests of free and equal individuals. Against this background, the exercise of the right to vote is a purely personal manifestation of individual interests. The majority or minority after an election is merely a retrospectively established sum of articulated interests that randomly coincide. However, the sociological background of each individual vote—the party, the class, etc—remains beyond the law’s horizon. While Rousseau fought against the formation of parties, since they distort the expression of individual interests, until recently, constitutional law, at least, ignored the powerful parties that had in fact emerged. Parties appeared to be mere sociological entities that lacked legal personality. Legally, only individual voters existed. In this way, all areas of law were adjusted to the individualist and intellectualist type of human being. The old patriarchal thought of duty-based and duty-infused rights continued to languish in only one corner of the legal order: family law. It was still permissible to entrust the husband and father with rights *vis-à-vis* his wife and children in the expectation that he would exercise those rights dutifully. However, even in family law, legal guarantees were increasingly introduced to ensure that husbands and parents exercised their rights properly—consider, for example, the Juvenile Court Act^36^ or the Juvenile Welfare Act.^37^ In family law, too, the transformation of duty-infused rights into self-serving rights and into duties that benefit third parties was under way.

In the meantime, however, the fictional nature of the alleged empirical average type of the liberal legal era has become more and more apparent. By no means is the individual always able to recognise its own interests, or to pursue its interests, having recognised them. By no means is the individual always even motivated by its interests. In cases of recklessness and of inexperience in times of crisis, a law that is tailored towards a clever, free and interested individual fails those who do not possess those attributes. Usury laws reintroduced a trend towards protecting legal actors from themselves. The trend continues with respect to contractual limitations imposed by occupational safety legislation. Beginning in Austria, the principle of party autonomy in civil proceedings is increasingly displaced by a judge who helpfully intervenes in the dealings between the parties, on their behalf and in their own interests. In criminal law, Feuerbach’s deterrence-based rationale breaks down; it becomes apparent that the criminal human being is the one least able rationally to balance the costs and benefits of a particular course of action and then to choose the best alternative. Instead, it emerges that criminals must first be bettered—that is, they must be equipped with the ability to recognise their own interests and to follow exclusively their own well-understood interests. Behind the monolithic image of the offender, the betterment approach reveals a significantly more diverse range of legally relevant psychological types of offenders: occasional and habitual offenders, those who can be reformed and those who are incorrigible. The new school of criminal law can rightly call itself sociological criminal law, since it recognises the legal significance of a range of hitherto merely sociological circumstances. A new understanding of the individual is approaching; the turn of a legal area is looming; a new legal epoch begins.

Since the new image of an individual also considers the intellectual, economic and social status of a legal subject, it is much more realistic than the abstract models of the liberal era that were based on free, selfish and clever individuals. Henceforth, the legal human being is no longer Robinson or Adam, an isolated individual, but rather a human being who is part of a society, part of a collective [*ein Kollektivmensch*]. However, as the new legal type of human being begins to bear more resemblance to social reality, the legal subject also begins to divide into multiple socially, and now also legally, relevant types. This development can be illustrated particularly well with respect to labour law, which is leading the way into the social legal era, just as commercial law led the way into the liberal era.

Private law, the ‘civil’ law, knows only equal legal subjects, who enter into contracts based on their free will—and not the worker, relatively inferior in power *vis-à-vis* the employer. Private law also knows nothing of the solidarity amongst workers that attempts to compensate for the inferiority of the individual worker towards the employer, and nothing of the associations that, based on collective agreements, are the actual parties to the employment contract. Private law exclusively sees the individual parties and the individual employment contract. Finally, it knows nothing of the unity of a particular business: private law merely recognises a multiplicity of employment contracts with employees who are legally entirely unrelated to one another. It does not see the workforce of a business as a connected sociological unit. It cannot see the forest for the trees.

By comparison, the key characteristic of labour law is its proximity to real life. Unlike the abstract civil law, it does not see merely persons, but rather employers, workers and employees; not simply individuals, but associations and businesses; not just free contracts, but also the difficult economic power struggles that form the background to these allegedly consensual contracts. Labour law sees individuals as limbs of their associations, of their businesses and, ultimately, of the whole economy and society, with all the motives that this entails: motives of community spirit, or at least motives of that extended self-interest that is called solidarity. Soon, public law, too, may embrace this new image of the individual. We are in the midst of rethinking the term democracy: democracy is no longer predicated on the isolated individual, but rather on the image of an individual who forms part of a collective. Democracy is no longer about the ‘equality of all human beings’, but rather about the selection of a leader. In this sense, democracy no longer refers to a sum of individuals, but rather to a significantly more complicated sociological whole, consisting of social groups, classes and parties. This applies not only to the sociological and political concept of democracy, but also to the legal concept of democracy: proportional representation grants such groups legal significance. Whereas parties used to form part of the scenery, they are now entering the stage of constitutional law, as important state organs.

However, to think of the individual as a part of a collective also entails thinking of the individual as incorporating parts of a collective ethos. A new ‘ethicisation’ of law is under way. Law is infused with a sense of ethical duty: it is said that ‘property entails obligations’ and that ‘the right to vote entails the duty to vote’. Jhering already elevated the struggle for law into a moral obligation. By recognising this duty-infused nature of law, the social legal era returns to the thought of the patriarchal legal era: every right appears to us, too, as merely a fief of the collective whole. However, compared to the patriarchal legal era, rights are not merely infused with duties, but also conditioned by concepts of dutiful exercise. The wartime economy accustomed us to perceiving every right as merely a provisional right, granted to an individual until further notice, in the expectation of its dutiful exercise. When social groups do not dutifully exercise their rights, the legislator stands ready at all times to withdraw rights that have been abused. All rights have become subject to revocation.

This sketch of the different conceptions of the individual as the object of a legal order does not, however, exhaust our subject. It remains to sketch, in even larger strokes, how the law imagined the individual as its subject, as its creator. Since human legislators are not self-evident but a recent achievement of history, one must consider whether law ever imagined that it would be authored by individual human beings.

In primeval Germanic times, law, customs, morality and religion were one and the same. Law was at the same time the wisdom of the ancestors, the voice of the people’s conscience and the will of the gods—and, as such, beyond the reach of individual human legislators. Even when legal codes and *Weistümer* changed the law, progressive developments were disguised as continuations of pre-existing law. In the introduction to the *Sachsenspiegel*, it still said: ‘I did not invent this law myself; it has been handed down to us by our benevolent ancestors.’^38^

Thus, the acts of the first human legislators must have been perceived as sacrilegious interferences with the prerogative of the gods. In Germany, the transition to human legislation occurred particularly late and particularly slowly. The laws of the Merovingians and the Carolingians were a decisive turning point. The king was not yet able to issue laws directly to the people, but he was able to command his civil servants. Statutory law was merely magisterial law [Amtsrecht, *ius honorarium*] and this magisterial law bound only the royal courts. The people and the people’s courts continued to be governed by traditional customary law. The struggle for domination between magisterial law and the people’s law continued for a long time and is one of the various manifestations of the ‘eternal process between law and the state’.

Against this background, we now jump directly to the modern era. Even well into the modern era, legal science and legal practice do not simply rely on statute, but invoke various other authorities in support: the Bible or the classical writers of antiquity. This shows that the contemporary unconditional obligatoriness of the state’s laws was not yet known. In the era of natural law, the validity of positive law depended not on its proper enactment by the state authorities, but on the correct substantive content of the law. In light of this position, Hobbes still had to emphasise ‘that law is not counsel, but command’.^39^

The reception of Roman law, the written law of the Roman Emperor, who was considered to be a forefather of the German Reich, prepared the way for the acceptance of the will of the state as law. However, only in the absolute state was the equation of the will of the state with law achieved. Magisterial law prevails only in a state governed by magistrates. It is only during the Enlightenment that the utility-driven will of the legislator displaces the impulse-driven nature of the people’s spirit. This changing awareness is best reflected in language. Modern legal language appears almost immediately. It is the language of a state that has become aware of its power to legislate, the wonderfully consistent language of the categorical command that increasingly distances itself from a language that seeks to persuade, to convince or to lecture. Finally, the human legislator enters the stage of history, in the shape of the absolute ruler.

The gradual shift from an absolute to a constitutional state entails that the will of the state begins to incorporate the will of the people, and that the law is, once again, depersonalised and collectivised. Now, legislation does not merely refer back to the people’s deputies, but to the people as a whole. Increasingly, experts and interested stakeholders are consulted when legislation is prepared. Sometimes this occurs informally, sometimes—in the case of the Economic Council of the Reich—it occurs in accordance with constitutional provisions. The law turns into a new kind of people’s law—a law no longer shaped by an unorganised, impulse-driven spirit of the people, but by an organised, utility-driven will of the people.

Thus, the course of history runs from the unconscious collective will, via the conscious individual will, to the conscious collective will embodied by the legislator. In this way, the development of the individual human being as a legal subject corresponds to the individual human being as a legal object. Initially, all law is subjectively and objectively collective law, the law reflecting a collective awareness of human beings forming part of a collective. Next, law becomes individualised in both respects: the law of an individual legislator for entirely unrelated individuals. Ultimately, law is in both senses being collectivised again—not as a patriarchal law, but as the law of an organised community.

And in this way, we return once more to the individual as the legal object. The thoughts offered here in this regard are by no means without precedent. In different forms, and hidden behind a transparent veil of historical constructions, they have always been considered by teachings on the state of nature. The state of nature is essentially the original state of the human soul, from which the law departs. Throughout time, different legal eras alternated between courting two opposing understandings of the state of the soul, which the teachings on the state of nature respectively labelled ‘appetitus societatis’ (Grotius)^40^ and ‘homo homini lupus’ (Hobbes).^41^ In his brilliant and rich lecture,^42^ Georg Jellinek has shown how the old theory of the state departed from a human archetype that resembled the image of humanity’s historic progenitor: the old Adam, whose forms of appearance change throughout history—that is, the individual human being in the law.

Mentioning Jellinek’s name brings back memories of one of the most glorious times of our glorious university: the Heidelberg of the Georg Jellineks, Wilhelm Windelbands, Emil Lasks, Ernst Tröltschs, Eberhard Gotheins and Max Webers. To this Heidelberg I owe my intellectual heritage. Now that I have the privilege to return to the old homeland of my spirit, I know of no better vow than a pledge to these grand masters. However, by a strange coincidence, two other great persons have also found not their sphere of influence, but their final resting place in Heidelberg. Two men who were to me, in the two fields of my working life and in an intimate sense, teachers, masters and fatherly friends: Franz von Liszt and Friedrich Ebert. Let me also mention their names at this solemn moment, with never-ending admiration and gratitude. I will plough this new field in their spirit.

*Schaff, das Tagwerk meiner Hände, hohes Glück, daß ichs vollende!*

Fortune, now these hands replenish

With fresh strength their toil to finish!^43^

## Footnotes

1 ‘Einigung mit der SPD’ *Heidelberger Tageblatt* (Heidelberg, 13 November 1926) 1; ‘Wieder große Koalition im Reich’ *Heidelberger Neueste Nachrichten* (Heidelberg, 13 November 1926) 1.

2 ‘Die Unterredung Hoesch-Briand: Nicht ungünstig verlaufen’ *Heidelberger Neueste Nachrichten* (Heidelberg, 13 November 1926) 1.

3 ‘Der Femeausschuß wieder in Berlin: Beginn der Zeugenvernehmungen’ *Deutsche Allgemeine Zeitung* (Berlin, 13 November 1926) 1. See also ‘“Black Reichswehr” Trials’ *The Times* (London, 12 November 1923) 13.

4 Gustav Radbruch, ‘Abriß meines Lebens’ in Gustav Radbruch, *Gesamtausgabe*, vol 16: *Biographische Schriften* (Arthur Kaufmann ed, CF Müller 1988) 279.

5 Gustav Radbruch, *Grundzüge der Rechtsphilosophie* (Quelle & Meyer 1914). Radbruch substantially reworked the monograph, renamed it and republished it in 1932: Gustav Radbruch, *Rechtsphilosophie* (3rd edn, Quelle & Meyer 1932). The 1932 edition is the basis of the work’s translation by Kurt Wilk: Gustav Radbruch, ‘Legal Philosophy’ in Kurt Wilk (tr), *The Legal Philosophies of Lask, Radbruch, and Dabin* (Harvard UP 1950).

6 Gustav Radbruch, ‘Gesetzliches Unrecht und übergesetzliches Recht’ [1946] Süddeutsche Juristenzeitung 105. An English translation of the text appeared in this journal as Gustav Radbruch, ‘Statutory Lawlessness and Supra-Statutory Law’ (2006) 26 Oxford Journal of Legal Studies 1.

7 HLA Hart, ‘Positivism and the Separation of Law and Morals’ (1957) 71 Harv L Rev 593; Lon L Fuller, ‘Positivism and Fidelity to Law: A Reply to Professor Hart’ (1958) 71 Harv L Rev 630. See also Stanley L Paulson, ‘Lon L Fuller, Gustav Radbruch, and the “Positivist” Theses’ (1994) 13 Law and Philosophy 313.

8 Compare Hart (n 7) 617–18 with Fuller (n 7) 636–7.

9 Heinrich Stoll, ‘Der Mensch im Recht von Gustav Radbruch’ (1928) 128 Archiv für die civilistische Praxis 121, 121.

10 Anton-Hermann Chroust, ‘The Philosophy of Law of Gustav Radbruch’ (1944) 53 The Philosophical Review 23, 24; Edwin Kuntz, ‘Gustav Radbruchs Abschied von seinem Lehrstuhl’ *Rhein-Neckar-Zeitung* (Heidelberg, 15 July 1948) 2; Adolf Laufs, ‘Gustav Radbruch—Leben und Werk’ in *Heidelberger Jahrbücher* (Springer 1979) 78.

11 Arthur Kaufmann, ‘Gustav Radbruch—Leben und Werk’ in Gustav Radbruch, *Gesamtausgabe*, vol 1: *Rechtsphilosophie* (Arthur Kaufmann ed, CF Müller 1987) 50–1.

12 Georg Jellinek, ‘Adam in der Staatslehre’, speech delivered before the Heidelberg Association of History and Philosophy (1893), subsequently reprinted in Georg Jellinek, *Ausgewählte Schriften und Reden von Georg Jellinek* (Häring 1911) vol 2.

13 Alain Supiot, *Homo Juridicus: On the Anthropological Function of the Law* (Verso 2007) ix.

14 Here Radbruch’s thoughts resemble those of Adorno with respect to the position of the human in history. See Theodor Adorno, *Negative Dialektik* (Suhrkamp 1966) 297.

15 Radbruch offers a more substantive critique of the way in which a legal order reduces individuals to a composition of legally relevant facts in his *Rechtsphilosophie*. See Radbruch, *Rechtsphilosophie* (n 5) 98–100.

16 For two recent contributions to the debate on the reasonable person, see Mayo Moran, *Rethinking the Reasonable Person: An Egalitarian Reconstruction of the Objective Standard* (OUP 2003) and John Gardner, ‘The Many Faces of the Reasonable Person’ [2015] LQR 563.

17 Radbruch repeats this claim, more forcefully, a few years later: Gustav Radbruch, ‘Vom individualistischen zum sozialen Recht’ (1930) 12 Hanseatische Rechts- und Gerichtszeitschrift 457. The article was reprinted in Gustav Radbruch, *Gesamtausgabe*, vol 2: *Rechtsphilosophie II* (Arthur Kaufmann ed, CF Müller 1993) 485–95.

18 See Radbruch, *Gesamtausgabe* (n 21) 486.

19 Stoll (n 9) 122; Walter Jellinek, ‘Abschied von Gustav Radbruch’ (1949) 4 Deutsche Rechts-Zeitschrift 565, 566.

20 Kaufmann (n 11) 36.

21 Kaufmann (n 11) 50–1.

22 See Radbruch, *Rechtsphilosophie* (n 5) 98–100. These principles subsequently resurface in Radbruch, ‘Gesetzliches Unrecht und übergesetzliches Recht’ (n 6) 107.

23 HLA Hart, *The Concept of Law* (2nd edn, Clarendon Press 1994) 117.

24 Radbruch, ‘Vom individualistischen zum sozialen Recht’ (n 21) 486.

25 See eg Kuntz (n 10) 2; Jellinek (n 23) 566; Laufs (n 10) 78; Alexander Hollerbach, ‘Notizen zu Radbruchs “Vorschule der Rechtsphilosophie”’ in Manfred Seebode (ed), *Festschrift für Günter Spendel zum 70. Geburstag am 11. Juli 1992* (De Gruyter 1992) 150.

26 Hollerbach (n 29) 150 (translator’s translation).

27 ‘Professor Gustav Radbruch’ *The Times* (London, 31 December 1949) 7.

28 ‘Der Mensch im Recht’, inaugural lecture delivered by Gustav Radbruch at Heidelberg University on 13 November 1926. The lecture’s first printed edition was dedicated to the 50th anniversary of the German *Reichsjustizministerium* (Imperial Ministry of Justice) and appeared in *Recht und Staat in Geschichte und Gegenwart* (1927) 5–18. An edited version of the lecture was reprinted *inter alia* in Gustav Radbruch, *Gesamtausgabe* (n 21) 467–76. The translation here is published with permission of CF Müller Juristischer Verlag Heidelberg. All references were added by the editors of the German original and by the translator.

29 Max Stirner, *Der Einzige und sein Eigentum* (Otto Wigand 1845).

30 Rudolf von Jhering, *Der Kampf um’s Recht* (4th edn, Verlag der Manz’schen Buchhandlung 1874).

31 The reception of Roman law.

32 In contrast to the modern, authoritarian connotations of the term ‘Police State’, the expression is used here in a ‘specifically German’ sense to refer to the general internal administration of the state from the 17th to the 18th century. See Hans Maier, *Die ältere deutsche Staats- und Verwaltungslehre* (CH Beck 2009) 13, 23.

33 Immanuel Kant, *Perpetual Peace* (M Campbell Smith tr, 3rd edn, Macmillan 1917) 153–4: ‘The problem of the formation of the state, hard as it may sound, is not insoluble, even for a race of devils, granted that they have intelligence. It may be put thus: – “Given a multitude of rational beings who, in a body, require general laws for their own preservation, but each of whom, as an individual, is secretly inclined to exempt himself from this restraint: how are we to order their affairs and how establish for them a constitution such that, although their private dispositions may be really antagonistic, they may yet so act as a check upon one another, that, in their public relations, the effect is the same as if they had no such evil sentiments.”’

34 Niccolò Machiavelli, *Discourses on Livy* (1531) bk I, ch 3.

35 Johann Anselm von Feuerbach, *Lehrbuch des gemeinen in Deutschland gültigen peinlichen Rechts* (Heyer 1847).

36 Jugendgerichtsgesetz of 16 February 1923, 1923 Reichsgesetzblatt I, 135.

37 Reichsgesetz für Jugendwohlfahrt of 9 July 1922, 1922 Reichsgesetzblatt I, 633.

38 Maria Dobozy, ‘Introduction’ in Maria Dobozy (ed), *The Saxon Mirror: A Sachsenspiegel of the Fourteenth Century* (University of Pennsylvania Press 1999) 13.

39 Thomas Hobbes, *Leviathan* (first published 1651, Richard Tuck ed, CUP 1996) 183.

40 Hugo Grotius, *De iure belli ac pacis* (1680), prolegomena, para 6.

41 Thomas Hobbes, *Elementa philosophica de cive* (1657) 10.

42 Jellinek (n 13).

43 This translation of Goethe’s poem ‘Hoffnung’ is borrowed from Johann Wolfgang Goethe and Johann Christoph Friedrich Schiller, *Select Minor Poems of Goethe and Schiller* (John S Dwight tr, Hillard, Gray, and Co 1839) 34.

## Notes

Christian Neumeier, Laila Schestag, Benjamin Spagnolo, Matthew Windsor, and three anonymous reviewers commented on drafts of the translation and its introduction. The translator is very grateful for their feedback.

